# Global Distribution of Panton-Valentine Leukocidin–positive Methicillin-resistant *Staphylococcus aureus,* 2006

**DOI:** 10.3201/eid1304.061316

**Published:** 2007-04

**Authors:** Anne Tristan, Michele Bes, Helene Meugnier, Gerard Lina, Bülent Bozdogan, Patrice Courvalin, Marie-Elisabeth Reverdy, Mark C. Enright, François Vandenesch, Jerome Etienne

**Affiliations:** * INSERM, Lyon, France; † Unité des Agents Antibactériens, Centre National de Référence des Antibiotiques, Institut Pasteur, Paris; ‡ Imperial College London, London, England, United Kingdom

**Keywords:** community-acquired methicillin-resistant *Staphylococcus aureus*, *spa* typing, toxin, antibiotic resistance, Panton-Valentine leukocidin, staphylococcal chromosomal cassette *mec* element, multilocus sequence type, research

## Abstract

We determined the *agr* type, multilocus sequence type, protein A gene type (*spa* typing), toxin gene profile, and antimicrobial drug resistance profile of 469 isolates of Panton-Valentine leukocidin–positive community-acquired methicillin-resistant *Staphylococcus aureus* isolates (PVL-positive CA-MRSA). The isolates had been collected from around the world from 1999 through 2005 by the French National Reference Center for Staphylococci. We found that some continent-specific clones described in 2003, such as clone ST8, have now spread all over the world. Likewise, some PVL-positive CA-MRSA have spread to several countries on various continents. New clones have emerged (e.g., ST377) on new genetic backgrounds. PVL-positive CA-MRSA that were usually susceptible to most antistaphylococcal antimicrobial agents have acquired new resistance determinants (e.g., to gentamicin) in certain countries. The major trait shared by all these clones is a short staphylococcal chromosomal cassette *mec* element of type IV or V.

## Abstract 2

Some formerly continent-specific clones have now spread around the world

By definition, community-acquired methicillin-resistant *Staphylococcus aureus* (CA-MRSA) strains infect patients with no risk factors for acquiring an MRSA strain of hospital origin. CA-MRSA infections usually affect previously healthy young patients (*1*). These infections are mostly of skin and soft tissue, but deep-seated infections such as necrotizing pneumonia and disseminated invasive osteomyelitis have been described (*2*). Many CA-MRSA isolates produce Panton-Valentine leukocidin (PVL) and harbor a type IV staphylococcal chromosomal cassette *mec* (SCC*mec*) element, but some isolates harboring the SCC*mec* V element have been reported (*3*). PVL-positive CA-MRSA clones have spread throughout the world (*4*).

In 2003, Vandenesch et al. described continent-specific PVL-positive CA-MRSA clones (mainly on an *agr3* background) and characterized them by their sequence type (ST) (*4*). The main European clone, ST80, was detected in France, Switzerland, the Netherlands, England, Belgium, and Germany (*5*,*6*), but also in northern Europe (e.g., Denmark), where MRSA strains are rare in hospitals (*7*). One of the most prevalent PVL-positive CA-MRSA clones in the United States (USA300) belongs to ST8 (*8*); other US clones include USA400 (ST1), USA1000 (ST59), and USA1100 (ST30) (*9*). ST30 is also a major clone in Asia and Oceania (*10*,*11*) and is referred to as the South West Pacific clone (*11*). In Singapore, an international travel hub, several clones belonging to ST80, ST30, and ST59 have been reported (*12*). The prevalence of PVL-positive CA-MRSA varies considerably from continent to continent. In the United States, MRSA were isolated from 50% of patients with skin and soft-tissue infections seen in emergency departments of 11 cities (97% of isolates belonged to clone USA300) (*13*). In Europe, the prevalence is lower, at ≈1–3% (*14*).

Since 1999, the French National Center for Staphylococci has characterized 469 PVL-positive CA-MRSA isolates collected throughout the world. The isolates were typed by multilocus sequence type (MLST), protein A gene type (*spa* typing), antimicrobial drug resistance profiling, and toxin and resistance gene analysis. We describe the evolution and spread of PVL-positive CA-MRSA clones since their initial description.

## Materials and Methods

### Bacterial Isolates

From 1999 through 2005, the French National Reference Center for Staphylococci received 469 PVL-positive CA-MRSA isolates from 17 countries. These isolates were voluntarily sent to the center for PVL for gene detection and genomic characterization (clone designation). A single strain was selected per patient. Twenty-two isolates corresponded to an outbreak linked to a maternity unit that occurred in France in 2002–2003.

### DNA Extraction and Identification of *agr* Alleles

The strains were grown on brain–heart infusion agar or in brain-heart infusion broth at 37°C overnight. Genomic DNA was extracted with a standard procedure (*15*). Amplification of *gyr*A was used to confirm the quality of each DNA extract and the absence of PCR inhibitors. All PCR products were analyzed by electrophoresis on ethidium bromide–stained 1% agarose gels (Sigma, Lyon, France). The *agr* group (*agr* 1–4) was determined by PCR as previously described (*15*).

### Detection of the *mecA* Gene and SCC*mec* Typing

The *mec*A gene (coding for methicillin resistance) was detected by PCR as previously described (*3*). The staphylococcal chromosomal cassette *mec* (SCC*mec* I-IV) was detected as described by Oliveira et al. (*16*), and SCC*mec* type V was detected as previously described (*3*). The following reference strains were used as controls: COL (SCC*mec* I), BK2464 (SCC*mec* II), HU106 (SCC*mec* III), and BK2529 (SCC*mec* IV).

### Detection of Toxin Genes

Using PCR, we determined the presence of 22 specific staphylococcal virulence genes, as described previously (*15**,**17*). We detected sequences specific for staphylococcal enterotoxin genes (*sea-e, seh, sek, sem, seo*), as well as the toxic-shock syndrome toxin gene (*tst*), exfoliative toxin genes (*eta, etb, etd*), PVL genes (*luk*S-PV–*luk*F-PV), LukE–lukD leukocidin genes (*luk*E*–luk*D), the class F lukM leukocidin gene (*luk*M*)*, hemolysin genes (gamma [*hlg*], gamma variant [*hlgv*] and β [*hlb*]), and epidermal cell differentiation inhibitor genes (*edinA/B/C*).

### Antimicrobial Susceptibility Testing

MICs of penicillin, oxacillin, cefoxitin, kanamycin, tobramycin, gentamicin, erythromycin, clindamycin, tetracycline, pristinamycin, ofloxacin, fusidic acid, vancomycin, teicoplanin, fosfomycin, trimethoprim/sulfamethoxazole, rifampin, mupirocin, quinupristin/dalfopristin, and linezolid were determined by using the standard agar dilution technique, as recommended by the French Society for Microbiology. Structural genes for resistance to tetracycline, aminoglycosides, and macrolide-lincosamide-streptogramin (*18*) were identified by PCR. DNA was amplified in a model 2400 thermal cycler (Perkin-Elmer Cetus, Norwalk, CT, USA) with *Taq* (Qbiogene, Inc., Carlsbad, CA, USA) or *Pfu* (Stratagene, La Jolla, CA, USA) DNA polymerase, as recommended by the manufacturers. PCR elongation times and temperatures were adjusted to the expected size of the PCR product and to the nucleotide sequences of the primers, respectively.

### *spa* Typing

*spa* typing was performed on PVL-positive MRSA isolates as previously described (*19*). The x region of the *spa* gene was amplified by PCR. *spa* types were determined with the Ridom Staph Type software (Ridom GmbH, Würzburg, Germany), which automatically detects *spa* repeats and assigns a *spa* type according to Harmsen et al. (*20*) (http://spaserver.ridom.de). When the recently developed algorithm BURP (Based Upon Repeat Patterns) was applied, *spa* types were clustered into different groups with calculated cost between members of a group <6. *spa* types shorter than 3 repeats were excluded from analysis because no reliable deduction about ancestries can be made from these types. In addition to point mutation events, the new algorithm takes all other modifications that can occur (repeat or duplication or deletion) into account when the relatedness of different *spa* types is calculated. Because of speed constrains, a heuristic version of the EDSI-Alignment (Excisions, Duplications, Substitutions, Insertions), as described by Sammeth et al., was used (*21*). BURP *spa* clonal complexes (*spa*-CC) were automatically assigned by the Ridom Staph Type software (www.ridom.de/staphtype/).

### MLST

MLST was performed on representative strains of each clonal group, as described elsewhere (*22*). The allelic profile of each strain was obtained by sequencing internal fragments of 7 housekeeping genes (*arcC, aroE, glpF, gmk, pta, tpi, yqiL*) and entering them on the MLST home page (http://saureus.mlst.net), where 7 numbers depicting the allelic profile were assigned that defined a ST (*22*). Similar STs were grouped into clonal complexes (CC).

## Results

### *agr* and STs

The 469 PVL-positive CA-MRSA isolates were *agr* type 1 (n = 46, 9.8%), *agr*2 (n = 9, 1.9%), or *agr*3 (n = 414, 88.3%); none was *agr*4 (Table 1). The 469 PVL-positive isolates belonged to 11 STs: the *agr*1 isolates were ST8, ST59, ST22, ST766, or ST377; the *agr*2 isolates were all ST5; and the *agr*3 isolates were ST80, ST30, ST37, ST93, or ST1 (Table 1). None of the STs were shared by different *agr* types. The most frequent ST was ST80 (n = 357, 76.1%), which corresponded to the European clone.

**Table 1:** Geographic distribution of PVL-positive CA-MRSA clones according to their *agr*-type, ST, and *spa* type*

***agr*** **type**	**ST (CC)**	**No. (%)**	***spa* type** **(*spa* CC)**	**No. (%)**	**Location**		**Other reports in literature**
					**Detected before 2003**	**Newly detected (this work)**	
***agr*1**		**46 (9.8)**					
	**ST8 (8)**	**25 (54.3)**			**USA (** ***4*** **)**	**Netherlands, France, Spain,**	**N. Norway (** ***23*** **)**
						**Switzerland, French Polynesia**	**Greece (** ***24*** **)**
			**t008 (s)**	**25 (100.0)**			
	**ST59**	**7 (15.2)**			**USA (** ***4*** **)**	**Netherlands, France, Singapore**	**Taïwan (** ***25*** **)**
			**t437 (8)**	**6 (85.7)**			
			**t216 (8)**	**1 (14.3)**			
	**ST22 (22)**	**3 (6.5)**				**Netherlands, Germany**	
			**t005 (4)**	**2 (66.7)**			
			**t310 (4)**	**1 (33.3)**			
	**ST766 (22)**	**1 (2.2)**				**Singapore**	
			**t1276 (4)**	**1 (100.0)**			
	**ST377**	**10 (21.7)**				**Netherlands, France, Greece, Switzerland, Australia**	
			**t355 (6)**	**9 (90.0)**			
			**t595 (6)**	**1 (10.0)**			
***agr*2**		**9 (1.9)**					
	**ST5 (5)**	**9 (100.0)**				**France, Switzerland, Algeria**	
			**t311 (5)**	**5 (55.5)**			
			**t1277**	**3 (33.3)**			
			**t450**	**1 (11.1)**			
***agr*3**		**414 (88.3)**					
	**ST80**	**357 (83.2)**			**France, Switzerland (** ***4*** **)**	**Algeria, Singapore, Romania, Germany, Belgium, Greece, Slovenia, Netherlands**	**Denmark (** ***7*** **), Sweden (** ***7*** **), notheren Norway (** ***23*** **), England (** ***26*** **), Finland (** ***27*** **), Libya (** ***28*** **), Croatia (** ***29*** **), Scotland (** ***7*** **), Greece (** ***24*** **)**
			**t044 (1)**	**333 (93.3)**			
			**t131 (1)**	**9 (2.5)**			
			**t376 (1)**	**8 (2.2)**			
			**t639 (1)**	**2 (0.6)**			
			**t237 (1)**	**1 (0.3)**			
			**t1199 (1)**	**1 (0.3)**			
			**t1201 (1)**	**1 (0.3)**			
			**t1206 (1)**	**1 (0.3)**			
			**t1200 (†)**	**1 (0.3)**			
	**ST30 (30)**	**20 (4.8)**			**New Zealand, Western Samoa (** ***4*** **)**	**Netherlands, Australia, Japan, Switzerland, Singapore, China, French Polynesia**	**Sweden (** ***30*** **), Brazil (** ***31*** **), Uruguay (** ***32*** **), England (** ***26*** **), Hong Kong (** ***10*** **)**
			**t019 (2)**	**17 (75.0)**			
			**t021 (2)**	**1 (5.0)**			
			**t318 (2)**	**1 (5.0)**			
			**t1273 (2)**	**1 (5.0)**			
	**ST37 (30)**	**1 (0.2)**				**Netherlands**	
			**t914 (2)**	**1 (100.0)**			
	**ST93**	**4 (1.0)**			**Australia (** ***4*** **)**		
			**t202 (s)**	**4 (100.0)**			
	**ST1**	**32 (7.7)**			**USA (** ***4*** **)**	**France, Singapore**	**Switzerland (** ***28*** **), Canada (** ***33*** **)**
			**t128 (3)**	**18 (56.2)**			
			**t125 (3)**	**3 (9.4)**			
			**t558 (3)**	**1 (3.1)**			
			**t175 (7)**	**8 (25.0)**			
			**t1274 (7)**	**1 (3.1)**			
			**t1272 (s)**	**1 (3.1)**			

*PVL*,* Panton-Valentine leukodin; CA-MRSA, community-acquired methicillin-resistant *Staphylococcus aureus*; *agr*, accessory gene regulator; ST, sequence type; CC, clonal complexes; *spa* CC, *spa* clonal complexes. **†Excluded because <3 repeats; s, singleton.**

### *spa* Types and *spa* Clonal Complexes

The *spa* types were specific for *agr* type and ST. Minor variations of *spa* types (deletions or duplications of SSR units) were observed in several isolates within the same ST. For instance, 9 *spa* types were recognized among the 357 ST80 isolates, but t044 was the major *spa* type (n = 333, 93.3%); 8 of these *spa* types belonged to the same *spa* CC. A unique *spa* CC corresponded to each ST, except for ST1 isolates, which formed 3 different *spa* CC (Table 1).

### Geographic Origin and Spread

A previous study (*4*) showed a limited number of clones and a limited geographic distribution. Schematically, ST80 was detected in Europe, ST8 and ST1 in the United States, and ST30 in Oceania. The results of our study suggest intercontinental exchanges of several clones (Table 1): 1) the ST8 clone (USA300) from the United States toward Europe; 2) the ST1 clone (USA400) from the United States toward Europe and Asia; 3) the ST59 clone (USA1000) from the United States toward Asia; 4) the ST80 clone from Europe toward Asia (*12*); and 5) the ST30 clone from Oceania toward Europe. Countries with numerous international exchanges (e.g., Singapore) have the highest clonal diversity.

New clones have been detected since 2003. One, ST22, has been found in Europe only. Another new clone, ST766, which belongs to the same CC (CC22) as ST22, was found in Singapore (*12*). Clone ST377 (with a type V SCC*mec*) was reported simultaneously in Europe and Australia (*3*). Clone ST5 was detected in Europe only. Clone ST93 (the Queensland clone), described in Australia before 2003, has not yet been detected in other countries (*11*).

### Toxin Gene Content

The toxin gene distributions were compared to determine the genetic background of the different clones with minor variations. For instance, ST80 isolates were all positive for *etd*, *luk*S-PV, *luk*F-PV, and *edinA/B/C*; very few lacked *luk*DE or *hlgv* or harbored *hlB* (Table 2). Superantigenic toxin genes were detected in isolates that belonged to the different STs, except for ST377, ST80, and ST93 (Table 2).

**Table 2:** Toxin gene content of PVL-positive community-acquired methicillin-resistant *Staphylococcus aureus* clones*

*agr* type	ST	No. (%)	Toxin genes always detected (100%)	Toxin genes sometimes detected (%)
*agr1*		46 (9.8)		
	ST8	25 (54.3)	*lukPV, lukDE*	*hlgv* (95.8), *sek* (91.7), *sed* (16.7), *seb* (4.2), *hlB* (4.2)
	ST59	7 (15.2)	*lukPV, hlgv*	*hlB* (87.5), *sek* (87.5), *seb* (62.5), *lukDE* (12.5)
	ST22	3 (6.5)	*sem, seo, lukPV, hlg*	
	ST766	1 (2.2)	*sem, seo, lukPV, hlg*	
	ST 377	10 (21.7)	*lukPV, edinA/B/C, hlB, hlg*	
*agr2*		9 (1.9)		
	ST5	9 (100)	*sem, seo, lukPV, lukED, hlgv*	*edinA/B/C* (55.5)
*agr3*		414 (88.3)		
	ST80	357 (83.2)	*etd, lukPV, edinA/B/C*	*lukDE* (99.7), *hlgv* (99.7), *hlB* (0.8)
	ST30	20 (4.8)	*sem, seo, lukPV, hlg*	*sek* (5.0), *tst* (5.0)
	ST37	1 (0.2)	*sec, sem, seo, tst, lukPV, hlg*	
	ST93	4 (1)	*lukPV*	
	ST1	32 (7.7)	*lukPV, seh, lukDE, hlgv*	*sea* (78.1), *sec* (68.7), *sek* (68.7), *seb* (25.0), *edinA/B/C* (3.1)

*PVL, Panton-Valentine leukocidin; ST, sequence type; *luk*PV, PVL genes; *luk*DE, LukE–lukD leukocidin genes; gamma (*hlg*), gamma variant (*hlgv*), and β (*hlb)* hemolysin genes; *sea* to *see, seh, sek, sem, seo,* staphylococcal enterotoxin type A to E, H, K, M, and O genes, respectively; *tst,* toxic shock toxin gene; *eta, etb, etd*: exfoliative toxin type A, B, and D genes, respectively; *edinA/B/C,* epidermal cell differentiation inhibitor; *agr,* accessory gene regulator.

### Antimicrobial Drug Resistance

Isolates of each ST were grouped according to the number of antimicrobial drug resistance determinants they harbored. Initial PVL-positive CA-MRSA isolates were susceptible to most antimicrobial agents. For instance, 8 of the 25 ST8 isolates included in this study between 2003 through 2005 were resistant to penicillin and oxacillin alone, as were 17 of the 32 ST1 isolates and 18 of the 20 ST30 isolates (Appendix Table). ST80 isolates were initially resistant to penicillin, oxacillin, kanamycin, and tetracycline, and intermediate to fusidic acid. Since 2003, new antimicrobial resistance determinants have been acquired (e.g., gentamicin and ofloxacin). One ST8 isolate was resistant to penicillin, oxacillin, kanamycin, erythromycin, tetracycline, and ofloxacin; 1 ST1 isolate was resistant to penicillin, oxacillin, kanamycin, tobramycin, and gentamicin. A few ST80 isolates from Algeria were resistant to multiple antimicrobial agents. Most PVL-positive CA-MRSA strains with multiple antimicrobial resistant determinants were detected in Asia (Singapore, People’s Republic of China) or Africa (Algeria).

### Antimicrobial Resistance Genes

Antimicrobial resistance genes were sought in a subset of 153 ST80 isolates. The *aph3′-III* gene, which encodes high-level resistance to kanamycin and neomycin but also to amikacin and isepamycin, was detected in all 153 isolates (100%). The *tetK* efflux gene was detected in 125 (82%) of tetracycline-resistant isolates. The *ermC* gene, an erythromycin ribosome methylase, was detected in 61 (40%) of erythromycin-resistant isolates. The *far-1* gene was detected in 133 (87%) of fusidic acid–intermediate isolates.

### SCC*mec* Types

SCC*mec* type was determined for 22 *agr*1 isolates (10 ST8, 1 ST59, 1 ST22, and 10 ST377); 5 *agr*2 isolates (ST5); 190 *agr*3 isolates (179 ST80, 9 ST30, 2 ST93, and 7 ST1). All the isolates were SCC*mec* type IV, except for the 10 ST377 isolates, which were SCC*mec* type V.

## Discussion

This study has several findings. First, the continent-specific clones of PVL-positive CA-MRSA described in 2003 by Vandenesch et al. (*4*) have now spread to other continents. For instance, the ST1 clone USA400 is now detected in Europe and Asia. Some PVL-positive clones, such as ST1 and ST30, can now be considered pandemic, as they are detected in America, Europe, and Asia. Second, on a given continent, PVL-positive CA-MRSA have spread from country to country. For instance, in Europe, PVL-positive CA-MRSA were recently detected in Slovenia, Romania, and Croatia. Third, new PVL-positive CA-MRSA clones are emerging in strains with different genetic backgrounds. While most of the clones described in 2003 by Vandenesch et al. (*4*) had an *agr*3 background, the newly described clones are *agr*1 or *agr*2. Fourth, PVL-positive CA-MRSA, which were initially susceptible to most antistaphylococcal antimicrobial agents, have acquired new antimicrobial resistance determinants, to gentamicin and ofloxacin, for instance.

The global ST distribution of PVL-positive CA-MRSA isolates in this study depends, of course, on the sources of the isolates received by the French National Reference Center for Staphylococci and does not reflect the current epidemiology. Our collection represents a passive surveillance study and is related to the increased attention paid to CA-MRSA by certain regions. Nevertheless, our results agree with other reports which confirm that ST80 is mainly found in Europe (e.g., Denmark [*7*], Finland [*27*], and Greece [*24*]), but also in Libya (*28*); ST30 is pandemic (*34*).

PVL-negative, hospital-acquired MRSA belong to 5 major CCs (CC5, CC8, CC22, CC30, CC45). PVL-positive CA-MRSA of the same clonal classes were also detected in our study, with the exception of CC45, but the PVL + MRSA strains showed a broader CC diversity. For instance, none of the ST80 isolates belonged to CCs harboring hospital strains. PVL-positive CA-MRSA are gradually causing an increasing number of hospital-acquired infections in countries, such as the United States, where their prevalence is high (*35*). Kourbatova et al. reported that, during the period 2003–2004, five prosthetic joint infections were caused by USA300 strains (*36*).

The worldwide spread of PVL-positive CA-MRSA is likely related to international travel. ST80 isolates recovered in France were mainly detected in patients who were originally from Algeria, a country that reported a high rate of community- and hospital-acquired infections due to ST80 isolates in 2006 (*37*). Maier et al. recovered ST22 strains from Turkish migrants in Germany (*38*). Acquisition of new antimicrobial resistance determinants could be related to misuse of antimicrobial agents; the spread of multidrug-resistant strains could be facilitated by poor hygiene, regardless of country.

It is not known whether PVL-positive CA-MRSA clones arose through acquisition of the PVL phage by strains with a methicillin resistance background or, conversely, through acquisition of the SCC*mec* element by strains with a PVL-positive background. On analyzing the database of the French National Reference Center for Staphylococci, which contains >5,000 toxin gene profiles, we found isolates that were related to the PVL-positive MRSA clone ST80 but lacked either the PVL genes (5 isolates) or the *mec*A gene (7 isolates) (data not shown). These isolates, like the ST80 clone, were *agr*3, *etd*+, *edin*A/B/C+; 1 isolate (PVL– *mec*A+) was ST80, and another (PVL+ *mec*A–) was ST635 (a single-locus variant of ST80). These “atypical” isolates were discovered in Algeria, Switzerland, and France. We are unable to state whether they are ancestors or descendants of the most prevalent strains.

Deep-seated infections due to PVL-positive *S. aureus* can be extremely severe. For example, necrotizing pneumonia carries a mortality rate close to 75% (*39*). Whether the pathogenesis of these acute infections is related to the effect of PVL alone or in combination with other virulence factors such as superantigenic toxins is unclear. We found that some PVL-positive CA-MRSA clones (ST80) lacked any superantigenic toxin genes. Among the *S. aureus* virulence factors (not screened for here), ST30 strains harbor the *bbp* gene, which encodes bone sialoprotein (*34*). The SCC*mec* elements detected in our collection were type IV or V and corresponded to the smallest SCC*mec* element.

PVL-positive CA-MRSA are usually susceptible to most antistaphylococcal antimicrobial agents. Clone ST80 is usually resistant to tetracycline (mediated by the *tetK* gene), intermediate to fusidic acid (*far1* gene), and resistant to kanamycin (*aph3′*-*III* gene). We observed the emergence of rare isolates with multiple resistances to antimicrobial agents such as gentamicin and ofloxacin. From the therapeutic viewpoint, all the isolates were susceptible to trimethoprim-sulfamethoxazole, glycopeptides, and linezolid.

The involvement of PVL in CA-MRSA infections has not been proved in mouse sepsis and abscess models developed by Voyich: isogenic PVL-negative strains of USA300 and 400 were as lethal as wild-type strains, and they caused comparable skin diseases (*40*). Other experiments have to be conducted to determine if PVL is secreted in such a model.

In summary, since 2003 we have observed an impressive worldwide spread of PVL-positive CA-MRSA clones initially described at the beginning of this decade, and we have also detected PVL-positive CA-MRSA strains of other lineages. To counter this emerging global threat to public health, systematic surveillance of both hospital and community isolates is required, together with measures designed to limit their spread.

## Supplementary Material

Appendix Table Antimicrobial resistance profile of PVL-positive CA-MRSA clone*

## References

[1] Naimi TS, LeDell KH, Como-Sabetti K, Borchardt SM, Boxrud DJ, Etienne J, Comparison of community- and health care–associated methicillin-resistant *Staphylococcus aureus* infection. JAMA. 2003;290:2976–84. 10.1001/jama.290.22.2976 14665659

[2] Bocchini CE, Hulten KG, Mason EO Jr, Gonzalez BE, Hammerman WA, Kaplan SL. Panton-Valentine leukocidin genes are associated with enhanced inflammatory response and local disease in acute hematogenous *Staphylococcus aureus* osteomyelitis in children. Pediatrics. 2006;117:433–40. 10.1542/peds.2005-0566 16452363

[3] Garnier F, Tristan A, Francois B, Etienne J, Delage-Corre M, Martin C, Pneumonia and new methicillin-resistant *Staphylococcus aureus* clone. Emerg Infect Dis. 2006;12:498–500. 16704793 10.3201/eid1203.051040 PMC3291452

[4] Vandenesch F, Naimi T, Enright MC, Lina G, Nimmo GR, Heffernan H, Community-acquired methicillin-resistant *Staphylococcus aureus* carrying Panton-Valentine leukocidin genes: worldwide emergence. Emerg Infect Dis. 2003;9:978–84. 12967497 10.3201/eid0908.030089 PMC3020611

[5] Dufour P, Gillet Y, Bes M, Lina G, Vandenesch F, Floret D, Community-acquired methicillin-resistant *Staphylococcus aureus* infections in France: emergence of a single clone that produces Panton-Valentine leukocidin. Clin Infect Dis. 2002;35:819–24. 10.1086/342576 12228818

[6] Wannet WJ, Heck ME, Pluister GN, Spalburg E, van Santen MG, Huijsdans XW, Panton-Valentine leukocidin positive MRSA in 2003: the Dutch situation. Euro Surveill. 2004;9:28–9. 15591693

[7] Faria NA, Oliveira DC, Westh H, Monnet DL, Larsen AR, Skov R, Epidemiology of emerging methicillin-resistant *Staphylococcus aureus* (MRSA) in Denmark: a nationwide study in a country with low prevalence of MRSA infection. J Clin Microbiol. 2005;43:1836–42. 10.1128/JCM.43.4.1836-1842.2005 15815005 PMC1081382

[8] Roberts JC, Krueger RL, Peak KK, Veguilla W, Cannons AC, Amuso PT, Community-associated methicillin-resistant *Staphylococcus aureus* epidemic clone USA300 in isolates from Florida and Washington. J Clin Microbiol. 2006;44:225–6. 10.1128/JCM.44.1.225-226.2006 16390975 PMC1351968

[9] Pan ES, Diep BA, Charlebois ED, Auerswald C, Carleton HA, Sensabaugh GF, Population dynamics of nasal strains of methicillin-resistant *Staphylococcus aureus—*and their relation to community-associated disease activity. J Infect Dis. 2005;192:811–8. 10.1086/432072 16088830

[10] Ho PL, Cheung C, Mak GC, Tse CW, Ng TK, Cheung CH, Molecular epidemiology and household transmission of community-associated methicillin-resistant *Staphylococcus aureus* in Hong Kong. Diagn Microbiol Infect Dis. 2006;[Sep 19; Epub ahead of print] 10.1016/j.diagmicrobio.2006.08.003 16989976

[11] Vlack S, Cox L, Peleg AY, Canuto C, Stewart C, Conlon A, Carriage of methicillin-resistant *Staphylococcus aureus* in a Queensland Indigenous community. Med J Aust. 2006;184:556–9. 16768661 10.5694/j.1326-5377.2006.tb00379.x

[12] Hsu LY, Tristan A, Koh TH, Bes M, Etienne J, Kurup A, Community associated methicillin-resistant *Staphylococcus aureus*, Singapore. Emerg Infect Dis. 2005;11:341–2. 15759335 10.3201/eid1102.040782 PMC3320430

[13] Moran GJ, Krishnadasan A, Gorwitz RJ, Fosheim GE, McDougal LK, Carey RB, Methicillin-resistant *S. aureus* infections among patients in the emergency department. N Engl J Med. 2006;355:666–74. 10.1056/NEJMoa055356 16914702

[14] Del Giudice P, Blanc V, Durupt F, Bes M, Martinez JP, Counillon E, Emergence of two populations of methicillin-resistant *Staphylococcus aureus* with distinct epidemiological, clinical and biological features, isolated from patients with community-acquired skin infections. Br J Dermatol. 2006;154:118–24. 10.1111/j.1365-2133.2005.06910.x 16403104

[15] Jarraud S, Mougel C, Thioulouse J, Lina G, Meugnier H, Forey F, Relationships between *Staphylococcus aureus* genetic background, virulence factors, agr groups (alleles), and human disease. Infect Immun. 2002;70:631–41. 10.1128/IAI.70.2.631-641.2002 11796592 PMC127674

[16] Oliveira DC, de Lencastre H. Multiplex PCR strategy for rapid identification of structural types and variants of the mec element in methicillin-resistant *Staphylococcus aureus.* Antimicrob Agents Chemother. 2002;46:2155–61. 10.1128/AAC.46.7.2155-2161.2002 12069968 PMC127318

[17] Tristan A, Ying L, Bes M, Etienne J, Vandenesch F, Lina G. Use of multiplex PCR to identify *Staphylococcus aureus* adhesins involved in human hematogenous infections. J Clin Microbiol. 2003;41:4465–7. 10.1128/JCM.41.9.4465-4467.2003 12958296 PMC193818

[18] Strommenger B, Kettlitz C, Werner G, Witte W. Multiplex PCR assay for simultaneous detection of nine clinically relevant antibiotic resistance genes in *Staphylococcus aureus.* J Clin Microbiol. 2003;41:4089–94. 10.1128/JCM.41.9.4089-4094.2003 12958230 PMC193808

[19] Mellmann A, Friedrich AW, Rosenkotter N, Rothganger J, Karch H, Reintjes R, Automated DNA sequence-based early warning system for the detection of methicillin-resistant *Staphylococcus aureus* outbreaks. PLoS Med. 2006;3:e33. 10.1371/journal.pmed.0030033 16396609 PMC1325475

[20] Harmsen D, Claus H, Witte W, Rothganger J, Turnwald D, Vogel U. Typing of methicillin-resistant *Staphylococcus aureus* in a university hospital setting by using novel software for *spa* repeat determination and database management. J Clin Microbiol. 2003;41:5442–8. 10.1128/JCM.41.12.5442-5448.2003 14662923 PMC309029

[21] Sammeth M, Weiniger T, Harmsen D, Stoye J. Alignment of Tandem Repeats with Excision, Duplication, Substitution and Indels (EDSI). Proceedings of WABI, Mallorca, Spain, 2005 Oct 3–6. In: Casadio R, Myers G, editors. Algorithms in bioinformatics. Vol. 3692. New York: Springer-Verlag; 2005.

[22] Enright MC, Day NP, Davies CE, Peacock SJ, Spratt BG. Multilocus sequence typing for characterization of methicillin-resistant and methicillin-susceptible clones of *Staphylococcus aureus.* J Clin Microbiol. 2000;38:1008–15. 10698988 10.1128/jcm.38.3.1008-1015.2000 PMC86325

[23] Hanssen AM, Fossum A, Mikalsen J, Halvorsen DS, Bukholm G, Sollid JU. Dissemination of community-acquired methicillin-resistant *Staphylococcus aureus* clones in northern Norway: sequence types 8 and 80 predominate. J Clin Microbiol. 2005;43:2118–24. 10.1128/JCM.43.5.2118-2124.2005 15872230 PMC1153739

[24] Chini V, Petinaki E, Foka A, Paratiras S, Dimitracopoulos G, Spiliopoulou I. Spread of *Staphylococcus aureus* clinical isolates carrying Panton-Valentine leukocidin genes during a 3-year period in Greece. Clin Microbiol Infect. 2006;12:29–34. 10.1111/j.1469-0691.2005.01295.x 16460543

[25] Wang CC, Lo WT, Chu ML, Siu LK. Epidemiological typing of community-acquired methicillin-resistant *Staphylococcus aureus* isolates from children in Taiwan. Clin Infect Dis. 2004;39:481–7. 10.1086/422642 15356810

[26] Holmes A, Ganner M, McGuane S, Pitt TL, Cookson BD, Kearns AM. *Staphylococcus aureus* isolates carrying Panton-Valentine leucocidin genes in England and Wales: frequency, characterization, and association with clinical disease. J Clin Microbiol. 2005;43:2384–90. 10.1128/JCM.43.5.2384-2390.2005 15872271 PMC1153723

[27] Salmenlinna S, Lyytikainen O, Vuopio-Varkila J. Community-acquired methicillin-resistant *Staphylococcus aureus*, Finland. Emerg Infect Dis. 2002;8:602–7. 12023917 10.3201/eid0806.010313 PMC2738488

[28] Harbarth S, Francois P, Shrenzel J, Fankhauser-Rodriguez C, Hugonnet S, Koessler T, Community-associated methicillin-resistant *Staphylococcus aureus*, Switzerland. Emerg Infect Dis. 2005;11:962–5. 15963298 10.3201/eid1106.041308 PMC3367580

[29] Krzyszton-Russjan J, Tambic-Andrasevic A, Bukovski S, Sabat A, Hryniewicz W. First community-acquired methicillin-resistant *Staphylococcus aureus* (MRSA) strains in Croatia. Clin Microbiol Infect. 2006;12:697–8. 10.1111/j.1469-0691.2006.01493.x 16774575

[30] Miklasevics E, Haeggman S, Balode A, Sanchez B, Martinsons A, Olsson-Liljequist B, Report on the first PVL-positive community acquired MRSA strain in Latvia. Euro Surveill. 2004;9:29–30. 15591692

[31] Ribeiro A, Dias C, Silva-Carvalho MC, Berquo L, Ferreira FA, Santos RN, First report of infection with community-acquired methicillin-resistant *Staphylococcus aureus* in South America. J Clin Microbiol. 2005;43:1985–8. 10.1128/JCM.43.4.1985-1988.2005 15815039 PMC1081335

[32] Ma XX, Galiana A, Pedreira W, Mowszowicz M, Christophersen I, Machiavello S, Community-acquired methicillin-resistant *Staphylococcus aureus*, Uruguay. Emerg Infect Dis. 2005;11:973–6. 15963301 10.3201/eid1106.041059 PMC3367603

[33] Mulvey MR, MacDougall L, Cholin B, Horsman G, Fidyk M, Woods S. Community-associated methicillin-resistant *Staphylococcus aureus*, Canada. Emerg Infect Dis. 2005;11:844–50. 15963278 10.3201/eid1106.041146 PMC3367573

[34] Otsuka T, Saito K, Dohmae S, Takano T, Higuchi W, Takizawa Y, Key adhesin gene in community-acquired methicillin-resistant *Staphylococcus aureus.* Biochem Biophys Res Commun. 2006;346:1234–44. 10.1016/j.bbrc.2006.06.038 16806081

[35] Seybold U, Kourbatova EV, Johnson JG, Halvosa SJ, Wang YF, King MD, Emergence of community-associated methicillin-resistant *Staphylococcus aureus* USA300 genotype as a major cause of health care–associated blood stream infections. Clin Infect Dis. 2006;42:647–56. 10.1086/499815 16447110

[36] Kourbatova EV, Halvosa JS, King MD, Ray SM, White N, Blumberg HM. Emergence of community-associated methicillin-resistant *Staphylococcus aureus* USA 300 clone as a cause of health care–associated infections among patients with prosthetic joint infections. Am J Infect Control. 2005;33:385–91. 10.1016/j.ajic.2005.06.006 16153484

[37] Ramdani-Bouguessa N, Bes M, Meugnier H, Forey F, Reverdy ME, Lina G, Detection of methicillin-resistant *Staphylococcus aureus* strains resistant to multiple antibiotics and carrying the Panton-Valentine leukocidin genes in an Algiers hospital. Antimicrob Agents Chemother. 2006;50:1083–5. 10.1128/AAC.50.3.1083-1085.2006 16495274 PMC1426459

[38] Maier J, Melzl H, Reischl U, Drubel I, Witte W, Lehn N, H. Panton-Valentine leukocidin-positive methicillin-resistant *Staphylococcus aureus* in Germany associated with travel or foreign family origin. Eur J Clin Microbiol Infect Dis. 2005;24:637–9. 10.1007/s10096-005-0008-8 16167136

[39] Gillet Y, Issartel B, Vanhems P, Fournet JC, Lina G, Bes M, Association between *Staphylococcus aureus* strains carrying gene for Panton-Valentine leukocidin and highly lethal necrotising pneumonia in young immunocompetent patients. Lancet. 2002;359:753–9. 10.1016/S0140-6736(02)07877-7 11888586

[40] Voyich JM, Otto M, Mathema B, Braughton KR, Whitney AR, Welty D, Is Panton-Valentine leukocidin the major virulence determinant in community-associated methicillin-resistant *Staphylococcus aureus* disease? J Infect Dis. 2006;194:1761–70. 10.1086/509506 17109350

