# Supplementary material for: Global Distribution of Panton-Valentine Leukocidin–positive Methicillin-resistant Staphylococcus aureus, 2006
Source: Emerg Infect Dis. 2007 Apr;13(4):594–600. doi: 10.3201/eid1304.061316 (PMC2725977; doi:10.3201/eid1304.061316)
Supplement: Appendix Table — Antimicrobial resistance profile of PVL-positive CA-MRSA clone* [file 06-1316_appT-s1.pdf]

Publisher: CDC; Journal: Emerging Infectious Diseases
Article Type: Research; Volume: 13; Issue: 4; Year: 2007; Article ID: 06-1316
DOI: 10.3201/eid1304.061316; TOC Head: Research

Appendix Table. Antimicrobial resistance profile of PVL-positive CA-MRSA clone*

agr type	ST	N (%)	Antimicrobial resistance profile	N (%)	Countries of detection (N)
agr1		46 (9.8)			
	ST8	25 (54.3)	P, OX	8 (32.0)	Spain (2), Switzerland (2), United States (3), France (1)
			P, OX, K	1 (4.0)	Switzerland (1)
			P, OX, TE	3 (12.0)	Spain (1), the Netherlands (2)
			P, OX, K, E	6 (24.0)	France (1), the Netherlands (2), US (2)
			P, OX, E, OFL	1 (4.0)	France (1)
			P, OX, K, TE	1 (4.0)	French Polynesia (1)
			P, OX, K, E, OFL	1 (4.0)	US (1)
			P, OX, K, E, TE	1 (4.0)	Switzerland (1)
			P, OX, K, E, TE, OFL	1 (4.0)	Switzerland (1)
			P, OX, K, E, L, TE, MU	1 (4.0)	The Netherlands (1)
			P, OX, K, E, L, OFL, MU	1 (4.0)	United States (1)
	ST59	7 (15.2)	P, OX, K, E, L, TE	5 (71.4)	France (2), the Netherlands (2), Singapore (1)
			P, OX	1 (14.3)	United States (1)
			P, OX, K, T, G, E, L, TE	1 (14.3)	France (1)
	ST22	3 (6.5)	P, OX, K, T, G	2 (66.7)	The Netherlands (2)
			P, OX, FU	1 (33.3)	Germany (1)
	ST766	1 (2.2)	P, OX, K, T, G, E, TE, OFL	1 (100.0)	Singapore (1)
	ST377	10 (21.7)	P, OX, K, T, G	10 (100.0)	The Netherlands (1), France (1), Greece (5), Switzerland (2), Australia (1)
agr2		9 (1.9)			
	ST5	9 (100.0)	P, OX, TE, FU	8 (88.9)	France (3), Switzerland (5)
			P, OX, K, T, E, L, TE	1 (11.1)	Algeria (1)
agr3		414 (88.3)			
	ST80	357 (83.2)	P, OX, K	25 (7.0)	Algeria (9), France (13), Greece (1), Switzerland (2)
			P, OX, K,E	12 (3.4)	Algeria (5), France (6), Switzerland (1)
			P, OX, K, FU	19 (5.3)	Algeria (4), France (13), Switzerland (2)
			P, OX, K, TE	6 (1.7)	Algeria (1), France (5)
			P, OX, K, E, FU	8 (2.2)	Algeria (1), France (5), Switzerland (2)
			P, OX, K, E, L	1 (0.3)	France (1)
			P, OX, K, E, Rif	1 (0.3)	Algeria (1)
			P, OX, K, OFL, FU	1 (0.3)	Algeria (1)
			P, OX, K, TE, FU	205 (57.4)	Algeria (27), Belgium (1), France (147), Germany (1), Greece (3), The Netherlands (2), Slovenia (3), Switzerland (20), Singapore (1)
			P, OX, K, T, G	1 (0.3)	France (1)
			P, OX, K, E, L, FU	1 (0.3)	France (1)
			P, OX, K, E, TE, OFL	1 (0.3)	France (1)
			P, OX, K, E, TE, FU	59 (16.5)	Algeria (5), France (48), Romania (1), Switzerland (5)
			P, OX, K, E, L, TE, FU	2 (0.6)	France (2)
			P, OX, K, T, E, L, TE	1 (0.3)	Algeria (1)
			P, OX, K, T, G, OFL, FU	2 (0.6)	Algeria (2)
			P, OX, K, T, G, TE, FU	1 (0.3)	Algeria (1)
			P, OX, K, E, L, TE, OFL, FU	2 (0.6)	Algeria (2)
			P, OX, K, T, G, E, OFL, FU	1 (0.3)	Algeria (1)
			P, OX, K, T, E, L, OFL, FU	1 (0.3)	Algeria (1)
			P, OX, K, T, G, E, TE, FU	1 (0.3)	France (1)
			P, OX, K, T, G, OFL, FU, Rif	2 (0.6)	Algeria (2)
			P, OX, K, T, G, TE, FU, Rif	1 (0.3)	Algeria (1)
			P, OX, K, T, E, L, PRI, OFL, FU	2 (0.6)	Algeria (2)
			P, OX, K, T, G, E, L, PRI, OFL, FU	1 (0.3)	Algeria (1)
	ST30	20 (4.8)	P, OX	18 (90.0)	The Netherlands (1), Australia (8), Japan (1), New-Zealand (4), Western Samoa (1), Switzerland (2), Singapore (1)
			P, OX, K, T	1 (5.0)	French Polynesia (1)
			P, OX, K, T, G, E, L	1 (5.0)	China (1)
	ST37	1 (0.2)	P, OX, K, T, G, E, TE	1(100.0)	The Netherlands (1)
	ST93	4 (100.0)	P, OX	3 (75.0)	Australia (3)
			P, OX, E	1 (25.0)	Australia (1)
	ST1	32 (7.7)	P, OX	17 (53.1)	US (17)
			P, OX, E	10 (31.2)	US (9), France (1)
			P, OX, TE	4 (12.5)	United States (4)
			P, OX, K, T, G	1 (3.1)	Singapore (1)

Publisher: CDC; Journal: Emerging Infectious Diseases
Article Type: Research; Volume: 13; Issue: 4; Year: 2007; Article ID: 06-1316
DOI: 10.3201/eid1304.061316; TOC Head: Research

*Panton-Valentine leukocidin (PVL); CA-MRSA, community-acquired methicillin-resistant *Staphylococcus aureus*; penicillin (P), oxacillin, (OX), kanamycin (K), tobramycin (T), gentamicin (G), erythromycin (E), lincomycin (L), tetracycline (TE), pristinamycin (PRI), ofloxacin (OFL), fusidic acid (FU), rifampycin (Rif).
